# Human Papillomavirus Infection and *EGFR* Exon 20 Insertions in Sinonasal Inverted Papilloma and Squamous Cell Carcinoma

**DOI:** 10.3390/jpm13040657

**Published:** 2023-04-11

**Authors:** Hitoshi Hirakawa, Taro Ikegami, Norimoto Kise, Hidetoshi Kinjyo, Shunsuke Kondo, Shinya Agena, Narumi Hasegawa, Junko Kawakami, Hiroyuki Maeda, Mikio Suzuki

**Affiliations:** Department of Otorhinolaryngology, Head and Neck Surgery, Graduate School of Medicine, University of the Ryukyus, 207 Uehara, Nishihara 903-0215, Japan

**Keywords:** inverted papilloma, malignant transformation, human papillomavirus, epidermal growth receptor, mutation, phosphorylation

## Abstract

This study aimed to clarify the roles of high-risk human papillomavirus (HR-HPV) infection and epidermal growth factor receptor (EGFR) exon 20 mutations in sinonasal inverted papilloma (IP) and sinonasal squamous cell carcinoma (SNSCC). Samples were collected from 20 cases with IP, 7 with IP and squamous cell carcinoma (IP-SCC)*,* and 20 with SNSCC and examined for HPV infection and EGFR exon 20 mutations. Low- or high-risk HPV DNA was observed in 25% of IP, 57.1% of IP-SCC, and 35% of SNSCC cases. Transcriptionally active HR-HPV infections in IP-SCC and SNSCC, accompanied by p16 overexpression, were observed in 28.5% and 25% of cases, respectively. Heterozygous *EGFR* exon 20 amino acid insertions (ex20ins), located between amino acids 768–774, were observed in 45% of IP, 28.5% of IP-SCC, and 0% of SNSCC and chronic sinusitis cases. EGFR phosphorylation sites were located at tyrosine (Y) 845, Y1068, Y1086, and Y1197 and induced PI3K/AKT/mTOR activation. The phosphorylation pattern of EGFR with ex20ins resembled that of HPV-related SNSCC and oropharyngeal cancer. The transcriptionally active HR-HPV infection and ex20ins might be responsible for the pathogenesis of IP-SCC cases with different fashions. Since IP-SCC might be a multifactorial disease, further investigation is needed to understand IP-SCC etiology.

## 1. Introduction

Inverted Schneiderian papilloma (IP) is a benign neoplasm arising in the nasal cavity. Its etiology remains unclear, but several risk factors have been proposed, including human papillomavirus (HPV) infection and chronic inflammation [1,2]. A recent review revealed that 25–40% of cases with sinonasal papilloma have HPV infection [3].

In the clinical setting, IP sometimes accompanies synchronous and metachronous squamous cell carcinoma (IP-SCC). The rate of malignant transformation from IP to SCC has been estimated to be approximately 10% [3,4]. The detailed mechanism of malignant transformation is unclear, but several hypotheses have been proposed. The prevailing view is that high-risk (HR)-HPV infection might play a role; however, several contradictory results have been reported regarding HPV infection [5,6].

The molecular profiles of IP and IP-SCC suggest that mutations in epidermal growth factor receptor (EGFR) exon 20, which promote an active kinase conformation, especially exon 20 insertions (ex20ins), are candidate etiological factors [6,7,8,9,10,11]. EGFR is a transmembrane glycoprotein belonging to the ErbB family, which contains EGFR (also known as ErbB1/HER1), ErbB2 (HER2/neu), ErbB3 (HER3), and ErbB4 (HER4). EGFR activation leads to the stimulation of multiple intracellular signaling pathways, including PI3K/AKT and Jak-STAT. High EGFR expression and increased EGFR copy number account for 90% and 21% of cases of head and neck SCC, respectively; however, EGFR mutations are detected in only 1% of head and neck cancer cases [12].

Udager et al. first reported the presence of activating *EGFR* mutations in 88% of IP and 77% of IP-SCC cases, and this phenomenon might be distinct in IP and IP-malignant transformation among sinonasal squamous tumors [10]. Recent investigations also demonstrated that *EGFR* mutations, especially ex20ins, are frequently observed in IP and IP-sinonasal SCC (SNSCC) but not in SNSCC without IP lesions [6,13,14,15,16]. The significance of *EGFR* mutations in the etiology of IP and its malignant transformation remains unclear, especially ex20ins and the downstream phosphorylation targets of ex20ins. On the contrary, approximately 90% of *EGFR* mutations in NSCLC are observed within the tyrosine kinase domain, and they are the possible genetic mutation targets for treatment. *EGFR* exon 19 deletions and L858R point mutation in exon 21 are predominant *EGFR* mutations, representing the favorable response to EGFR tyrosine kinase inhibitors (TKIs). The ex20is has been reported as the subset of driver genes that account for 4% to 9% of NSCLC [17]. Although previous target therapies against the ex20ins in NSCLC were intensively investigated, the response rates and prognosis have been unsatisfactory compared to classical TKIs to other *EGFR* mutations [17]. Thus, it is important to clarify the status of the ex20ins in sinonasal lesions. Interestingly, several reports have suggested that *EGFR* mutations and HPV infection are mutually exclusive [6,9,18].

The present study investigated HR-HPV infection and *EGFR* exon 20 mutations to shed light on their roles in the pathogenesis of IP and IP-SCC.

## 2. Materials and Methods

### 2.1. Subjects

Samples were collected from the following patients at our hospital from 2008 to 2021: 20 with IP, 7 with IP-SCC, 20 with SNSCC, and 6 with chronic sinusitis as controls. These patients had no history of previous surgery, chemotherapy, or radiation therapy. SNSCC patients were independent from those with IP-SCC, and the SNSCC samples did not contain any IP part. All specimens were frozen immediately in liquid nitrogen after surgical excision or biopsy and stored at −80 °C until analysis.

This study was conducted with the approval of the Institutional Review Board of the University of Ryukyus (project identification code 156). Written informed consent was obtained from all participants prior to sample collection.

### 2.2. HPV Infection Analysis

HPV-related tumors for IP, IP-SCC, or SNSCC were defined as being positive for HPV DNA detected by PCR or DNA in situ hybridization (ISH) and p16 overexpression.

#### 2.2.1. Detection of HPV DNA, Measurement of Viral DNA Load, and Physical Status of HPV

Extracted DNA was subjected to PCR using the degenerate consensus primer sets MY09/MY11 and GP5+/GP6+, which were designed to amplify the *L1* region, as in previous studies [19,20]. We developed a new quantitative real-time PCR assay system for the *E6* and *E2* genes of HPV-18, -33, and -52 in addition to HPV-16 [19]. See the Supplementary Materials for details (Table S1).

#### 2.2.2. p16 Immunohistochemistry and ISH with HPV DNA Probes

Immunohistochemistry for p16^INK4a^ expression was performed with a CINTec^®^p16 Histology Kit (Roche Applied Science, Penzberg, Germany) [19]. The cutoff point for p16 overexpression was defined as diffuse (≥75%) tumor expression with at least moderate (+2/3) staining intensity, according to the 8th edition of the American Joint Committee on Cancer classification [21,22].

Because there were not enough frozen samples for PCR in three patients, DNAs extracted from formalin-fixed paraffin-embedded (FFPE) samples were used for PCR analysis; HPV-DNA ISH was used to confirm the presence of HR-HPV infection in these samples. Detailed methods for DNA extraction from FFPE samples are provided in the Supplementary Materials.

ISH of HR-HPV DNA was performed with a GenPoint HPV Biotinylated DNA Probe (Dako; Agilent Technologies, Inc., Santa Clara, CA, USA), which can detect HPV-16, -18, -31, -33, -35, -39, -45, -51, -52, -56, -58, -59, and -68 in FFPE sections, as described previously [18].

### 2.3. EGFR Mutation Analysis

#### 2.3.1. Sanger Sequencing of EGFR Exon 20

PCR was performed with *EGFR* exon 20-F and 20-R primers (0.24 μM each, Table S1) in a volume of 12.5 μL containing 6.3 μL GoTaq^®^ Green Master Mix (Promega, Madison, WI, USA) and 10 ng genomic DNA. See the Supplementary Materials for details.

#### 2.3.2. Screening of EGFR Phosphorylation Sites

A RayBio Human EGFR Phosphorylation Antibody Array 1 Assay (RayBiotech, Inc., Peachtree Corners, GA, USA) was used to screen specific EGFR phosphorylation sites (Y845, Y922, Y1045, Y1068, Y1086, Y1173, and Y1046/47) in two IP-SCC patients with ex20ins. ErbB2, ErbB3, and ErbB4 phosphorylation were also investigated simultaneously. See the Supplementary Materials for details.

#### 2.3.3. Western Blot Analysis

The phosphorylation of EGFR and EGFR-related pathway proteins in IP, IP-SCC, SNSCC, and chronic sinusitis with/without ex20ins were examined by western blotting. EGFR, phosphorylated (p)-EGFR (Y845, Y1068, Y1086, and Y1197), Akt, p-Akt (serine [S473]), 4E-BP1, p-4E-BP1 (threonine [T]37/46), STAT3, and p-STAT3 (Y705) were selected based on the RayBio Human EGFR Phosphorylation Antibody Array 1 results. See the Supplementary Materials for details.

#### 2.3.4. Immunohistochemistry for EGFR and p-EGFR

Four-micrometer-thick sections of FFPE samples were deparaffinized in xylene and hydrated in a graded alcohol series. Primary antibodies against EGFR (1:50 dilution, the same antibody used for western blotting) and p-EGFR (Y845, 1:50 dilution, the same antibody used for western blotting) were used. The immunohistochemical reaction was performed with a SAB-PO Kit (Nichirei Bioscience, Tokyo, Japan) [20].

### 2.4. Statistical Analysis

Statistical analysis was conducted with SPSS 25.0 (SPSS, Inc., Chicago, IL, USA). Pearson’s χ^2^-test was used for categorical data, and the Mann–Whitney U-test was used for continuous variables. *p*-values less than 0.05 were considered significant.

## 3. Results

### 3.1. Patient Characteristics (Table 1)

Sixteen (80%) of 20 IP cases had Krouse T3 or T4 stage. Although all cases underwent endoscopic sinus surgery, one (5%) recurred after surgery. In IP-SCC, the chief tumor location was the maxillary sinus (four cases, 57%), and there were four cases (57%) alive without disease. All SNSCC cases were advanced with stage III (36.4%) or stage IV (63.6%), and there were 13 cases (65%) alive without disease. There was no significant difference in the primary tumor site among the three groups.

**Table 1:** Patient characteristics.

Group		Number of Cases (%)
**IP**		*n* = 20
Sex	Male/female	16 (80)/4 (20)
Age, median (range)		57 (33–80) years
Krouse T classification	T1/T2/T3/T4	0/4/15/1
Primary tumor site	Maxillary sinus	14 (70.0)
	Ethmoid sinus	5 (25.0)
	Sphenoid sinus	1 (5.0)
Recurrence after surgery		1 (5%)
**IP-SCC**		*n* = 7
Sex	Male/female	4/3
Age, median (range)		57 (46–91) years
Primary tumor site	Maxillary sinus	4 (57.1)
	Ethmoid sinus	2 (28.6)
	Sphenoid sinus	1 (14.3)
Treatment	Surgery + CRT	2
	CRT/RT	3
	Other	2
Prognosis	Alive without disease	4
	Alive with disease	1
	Dead of disease	2
**SNSCC**		*n* = 20
Sex	Male/female	17 (93.3)/3(6.7)
Age, median (range)		59 (35–81) years
Primary tumor site	Maxillary sinus	17(85.0)
	Nasal cavity	2 (10.0)
	Ethmoid sinus	1 (5.0)
SCC subtype	Poor/moderate/well	2/11/7
Clinical T classification	T1/T2/T3/T4	0/0/7/13
Clinical N classification	N0/N1/N2/N3	12/5/3/0
UICC Stage	I/II/III/IV	0/0/6/14
Treatment	Total maxillectomy	1 (5.0)
	CRT/RT	17 (85.0)
	Other	2 (10.0)
Prognosis	Alive without disease	13 (65.0)
	Dead of disease	6 (30.0)
	Intercurrent disease death	1 (5.0)

CRT, concurrent chemoradiotherapy; IP, inverted papilloma; RT, radiation therapy; SCC, squamous cell carcinoma; SN, sinonasal; UICC, Union for International Cancer Control (7th edition).

### 3.2. HPV Infection Analysis

#### 3.2.1. HPV Infection in IP

Of the 20 patients with IP, HPV DNA was detected in 5 samples (25%) by PCR, 3 with HR-HPV (2 HPV-16 and 1 HPV-33), and 2 with low-risk HPV (HPV-6 and -11). However, the viral load could not be determined in the three HR-HPV cases because they were below the detection limit (Table 2).

**Figure 1:**
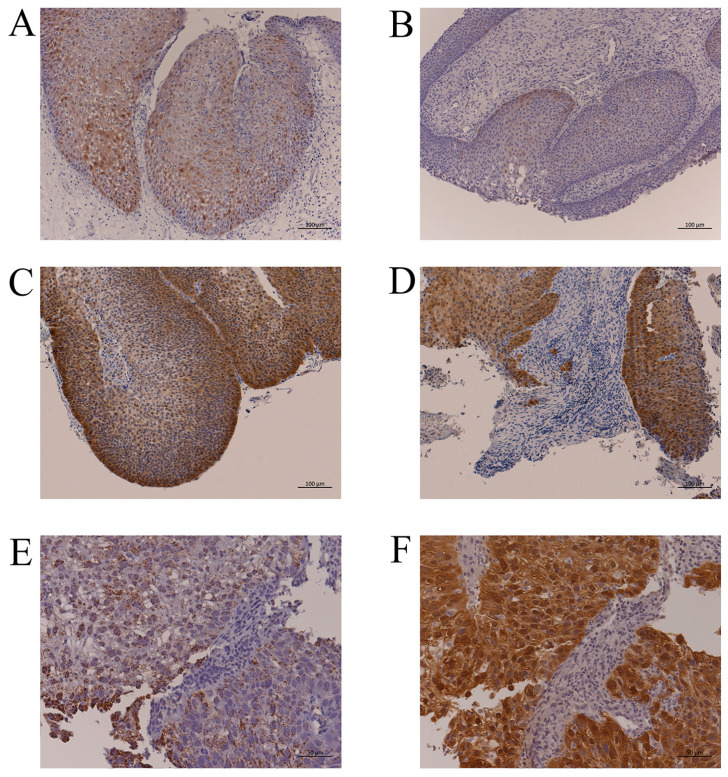
Representative p16 immunohistochemistry and HPV-DNA ISH. (**A**): IP without HPV infection (IP-2, Table 2). More than 75% of IP cells had p16 immunoreactivity, but the staining intensity was weak. Bar, 100 µm. (**B**): IP with HPV-16 DNA by PCR (IP-5, Table 2). There were few p16-positive cells despite HPV infection. Bar, 100 µm. (**C**): p16 overexpression in the IP part of IP-SCC with HPV-18 infection (IP-SCC-3, Table 2). Bar, 100 µm. (**D**): p16 overexpression in the SCC part of IP-SCC with HPV-18 infection (IP-SCC-3, Table 2). Strong p16 immunoreactivity was observed in SCC and IP. Bar, 100 µm. (**E**): HPV-DNA ISH of SNSCC with HR-HPV infection (SNSCC-5, Table 2). A positive reaction was observed in all cancer cells. Bar, 50 µm. (**F**): p16 overexpression in SNSCC with HR-HPV infection (SNSCC-5). Cancer cells showed strong p16 immunoreactivity. Bar, 50 µm.

The p16-positive cell rates in IP are shown in Table S2. Twenty-five percent of cases had ≥75% IP cells with p16 immunoreactivity; however, these cases had only weak staining, and there was no case with p16 overexpression (Figure 1A). There was also no significant relationship between p16 immunoreactivity and the presence of HR-HPV DNA (Table 2, Figure 1B). From these findings, no case was judged to have an HPV-related tumor.

#### 3.2.2. HPV Infection in IP-SCC

Of the seven patients with IP-SCC, four (57.1%) had HPV DNA: three with HR-HPV (HPV-16, -18, and -33) and one with low-risk HPV (HPV-11). The viral load of IP-SCC-1 (HPV-16) was low (Table 2), and p16 overexpression was not observed in this case. In the IP-SCC group, there were two cases with p16 overexpression (28.5%) who also had HR-HPV DNA by PCR and/or ISH.

The HPV-18-positive case (IP-SCC-3) had a viral load of 2.63 × 10^3^ copies/ng DNA and an *E2/E6* ratio of 8.0 × 10^−4^. In addition, p16 expression was moderate in the IP area (Figure 1C), whereas it was strong in the SCC area (Figure 1D). Thus, two IP-SCC patients (28.5%) were considered to have HPV-related tumors.

#### 3.2.3. HPV Infection in SNSCC

Of the 20 patients with SNSCC, 7 (35%) had HR-HPV DNA (Table 2). Although HPV-16 was detected in SNSCC-7 and -8, their viral loads were relatively low, and they did not overexpress p16. The remaining five HR-HPV-positive cases (SNSCC-2 to -6) also had p16 overexpression. Compared with the cases without p16 overexpression (0.7–1.58 copies/ng DNA), the p16 overexpression cases had a relatively high viral load (1.41 × 10^2^ to 1.63 × 10^6^ copies/ng DNA). All cases with HR-HPV DNA showed integration with an *E2/E6* ratio from 0 to 0.83. HPV-DNA ISH and p16 immunohistochemistry demonstrated that viral infection and p16 overexpression occurred in the same cancerous area (Figure 1E,F, SNSCC-5). Thus, HPV-related SNSCC was observed in 5 (25%) of 20 SNSCC cases.

### 3.3. Mutations in EGFR Exon 20

Heterozygous *EGFR* ex20ins were observed in nine IP cases (45%), two IP-SCC cases (28%), and zero SNSCC and chronic sinusitis cases (Table 3 and Table S3). A deduced amino acid substitution in *EGFR* was observed in five IP cases (25%), four IP-SCC cases (57.1%), one SNSCC case (4.5%), and zero chronic sinusitis cases (0%). EGFR ex20ins or substitutions were observed in 10 IP cases (50%), four IP-SCC cases (57.1%), and one SNSCC case (5%) (Table S3). There were significant differences in the frequency of these mutations between the IP and IP-SCC groups and the SNSCC group (*p* < 0.01, χ^2^ test).

Six different heterozygous *EGFR* ex20ins were observed in IP (Table 3, Figure S1), which were concentrated in the region encoding amino acids 768–774. Three cases had N771_H773dup, followed by two cases each with D770_N771insGF or H773_V774dup, and one case each with H773_V774insTH or S768_D770dup.

Two of the seven IP-SCC cases (28.6%) were heterozygous for *EGFR* ex20ins. Interestingly, the same mutation (S768_D770dup) was detected in both IP-SCC cases. Amino acid substitutions were also observed frequently in four of the seven IP-SCC cases (57.1%).

### 3.4. Screening of EGFR Phosphorylation Sites

**Figure 2:**
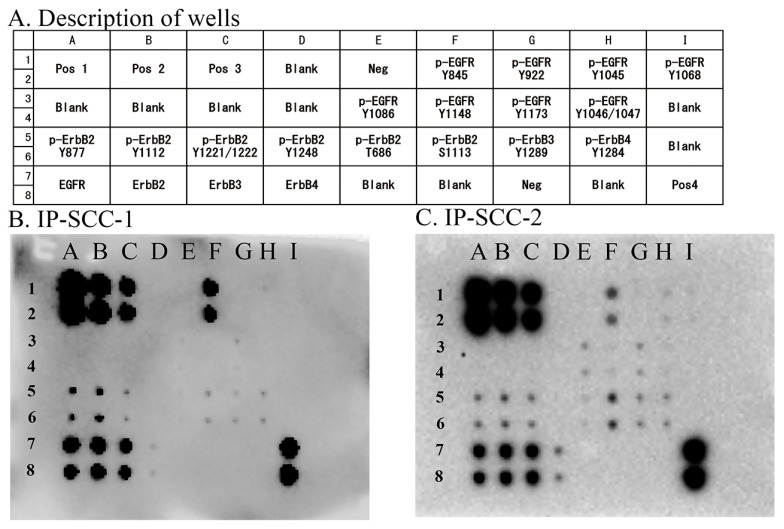
EGFR phosphorylation sites in IP-SCC. Eight phosphorylation sites in EGFR were investigated using an EGFR Phosphorylation Antibody Array 1 Assay. (**A**): Description of each well. (**B**): Results for IP-SCC1. (**C**): Results for IP-SCC-2. Of the eight sites, Y845 phosphorylation (F1 and F2 wells) was observed in both cases. Neg, negative control; Pos, positive control. Each antibody is spotted in duplicate vertically.

Eight EGFR phosphorylation sites were examined in the two IP-SCC cases carrying S768_D770dup. Of these phosphorylation sites, EGFR Y845 autophosphorylation was extensively observed in both cases (Figure 2). These cases also presented with ErbB2 (Y877, Y1112, Y1221/1222, T686, and S1113), ErbB3 (Y1289), and ErbB4 (Y1284) phosphorylation.

### 3.5. Western Blot Analysis

**Figure 3:**
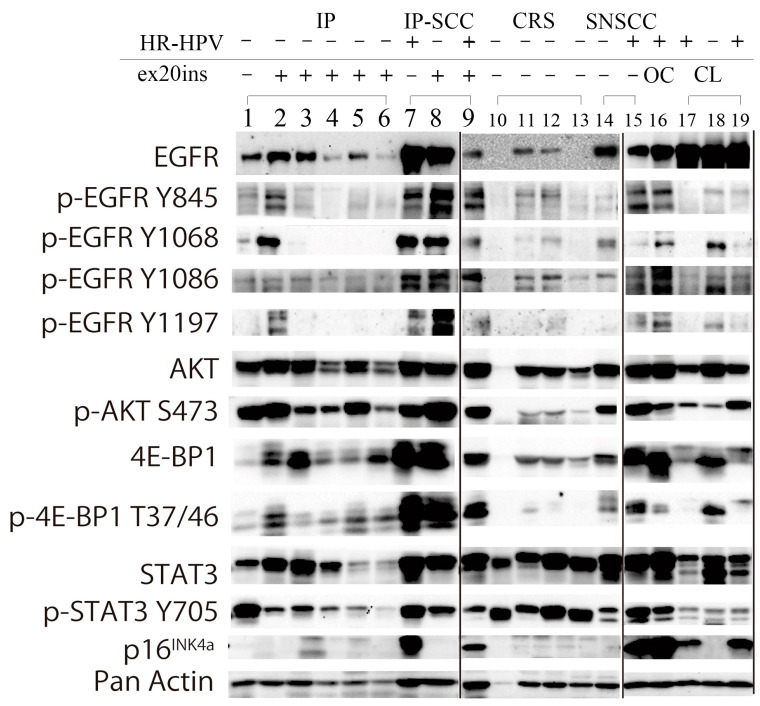
Western blot analysis of IP, IP-SCC, SNSCC, and chronic rhinosinusitis cases. EGFR and the related phosphorylation status of the signaling pathway induced by ex20ins and transcriptionally active HR-HPV infection (HR-HPV) were investigated by western blotting in IP (lanes 1–6), IP-SCC (lanes 7–9), chronic sinusitis (lanes 10–13), and SNSCC (lanes 14 and 15) cases. EGFR was expressed in all IP, IP-SCC, and SNSCC cases, and two of four sinusitis samples. AKT and 4E-BP1 were expressed in all IP, IP-SCC, SNSCC, and chronic sinusitis cases. The transcriptionally active HR-HPV-positive clinical samples (lanes 7, 9, 15, and 16) also demonstrated high p-EGFR, p-AKT, and p-4E-BP1 levels, regardless of ex20ins status. Lane 1: IP-19. Lane 2: IP-6 with S768_D770dup. Lane 3: IP-2 with D770_N771insGF. Lane 4: IP-1 with N771_H773dup. Lane 5: IP-7 with H773_V774insTH. Lane 6: IP-5 with H773_V774dup and HPV-16 DNA. Lane 7: IP-SCC-3 with HPV-18 DNA. Lane 8: IP-SCC-2 with S768_D770dup. Lane 9: IP-SCC-1 with S768_D770dup and HPV-16 DNA. Lane 10: chronic rhinosinusitis (CRS)-2. Lane 11: CRS-3. Lane 12: CRS-4. Lane 13: CRS-5. Lane 14: SNSCC-12 without ex20ins or HR-HPV DNA. Lane 15: SNSCC-2 with HPV-16 DNA. Lane 16: oropharyngeal carcinoma with HPV-16 DNA. Lane 17: UMSCC-47 cell line (a gift from Professor Thomas E. Carey, University of Michigan) derived from tongue carcinoma with HPV-16. Lane 18: SAS cell line (National Institute of Biomedical Innovation JCRB Cell Bank, Osaka, Japan) derived from tongue carcinoma without HPV infection. Lane 19: CaSki cell line (European Collection of Authenticated Cell Cultures, Salisbury, UK) derived from cervical cancer with HPV-16.

EGFR and the related phosphorylation status of the signaling pathway induced by ex20ins and HR-HPV infection were investigated by western blotting focusing on EGFR, p-EGFR (Y845, Y1068, Y1086, and Y1197), p-Akt (S473), p-4E-BP1 (T37/46), p-STAT3 (Y705), and p16^INK4a^ in IP, IP-SCC, chronic sinusitis, and SNSCC (Figure 3).

EGFR was expressed in all IP, IP-SCC, and SNSCC cases. Of the four EGFR phosphorylation sites, Y845 and Y1068 were robustly phosphorylated in IP-9 (lane 2) and IP-SCC-1 and -2 (lanes 8 and 9), who had S768_D770dup. IP-SCC-3 (lane 7), who had HR-HPV infection without ex20ins, demonstrated a similar EGFR phosphorylation pattern as cases with ex20ins. For ex20ins, only cases with S768_D770dup showed high p-EGFR levels (lanes 2, 8, and 9). IP cases without ex20ins (lane 1) had low p-EGFR and p-4E-BP1 levels but high p-AKT and p-STAT3 levels.

AKT (S473) was highly phosphorylated in all IP, IP-SCC, and SNSCC cases, regardless of HR-HPV infection and ex20ins status. 4E-BP1 (T37/46) was phosphorylated in all IP, IP-SCC, and SNSCC cases. p-4E-BP1 levels were much higher in IP-SCC (lanes 7–9) cases than in SNSCC and oropharyngeal carcinoma with HR-HPV infection cases. p-4E-BP1 levels were higher in IP with ex20ins cases than in IP without ex20ins (lane 1) and chronic sinusitis cases. STAT3 was expressed in all samples examined, while it was weakly expressed in IP-5 and IP-9 with H773_V774dup and H773_V774insTH, respectively. STAT3 (Y705) was similarly phosphorylated, and p-STAT3 levels were lower in IP cases than in chronic sinusitis cases.

HR-HPV-positive samples (lanes 7, 9, 15, and 16 in Figure 3) showed robust p16 expression in western blotting. The EGFR phosphorylation pattern of the HR-HPV-positive samples resembled that of the samples with ex20ins (lanes 2 and 8).

### 3.6. EGFR and p-EGFR (Y845) Immunohistochemistry

**Figure 4:**
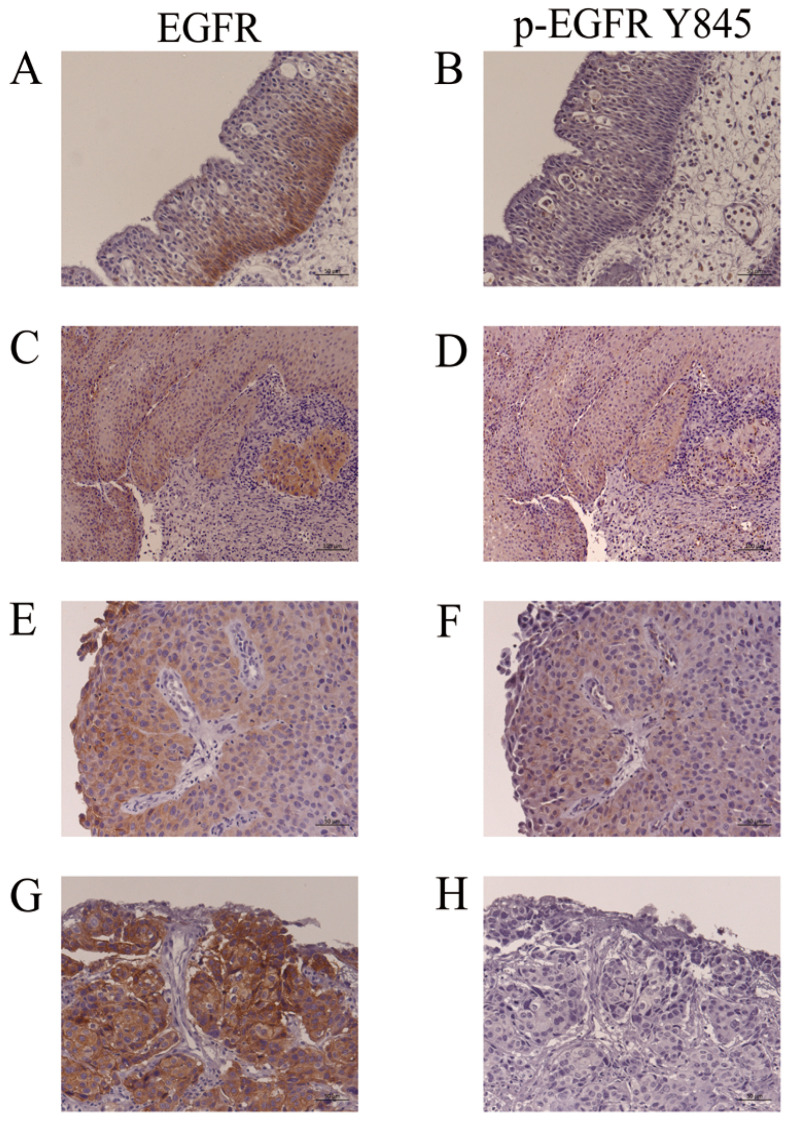
Immunohistochemistry for EGFR and p-EGFR (Y845). (**A**,**B**): IP-19. EGFR, but not p-EGFR (Y845), was detected in the basal cell layers of IP. (**C**,**D**): The IP part of IP-SCC-2 with S768_D770dup. (**E**,**F**): SCC part of IP-SCC-2 with S768_D770dup. EGFR and p-EGFR (Y845) were detected in the IP and SCC parts of IP-SCC-2. (**G**,**H**): SCC without HPV infection (SNSCC-12). There was intense EGFR staining (**G**) but none for p-EGFR (Y845) (**H**). Bar, 100 µm.

Representative findings of EGFR and p-EGFR (Y845) immunohistochemistry in IP (IP-19, Table 3), IP-SCC (IP-SCC-2, Table 3) and SNSCC (SNSCC-17, Table 3) cases are shown in Figure 4. EGFR expression was usually observed in the basal cell layer of IP (Figure 4A); however, p-EGFR (Y845) was not noted in IP (Figure 4B). IP-SCC cases with S768_D770dup showed EGFR and p-EGFR (Y845) immunoreactivity in the IP and SCC parts (Figure 4C–F). The SNSCC case with neither HR-HPV infection nor ex20ins demonstrated strong EGFR expression (Figure 4G) but not p-EGFR (Y845) (Figure 4H). These findings were consistent with the western blotting results.

## 4. Discussion

This study aimed to shed light on IP and IP-SCC in view of HR-HPV infection and *EGFR* exon 20 mutations.

HPV infection was observed in 25% of IP cases, 57.1% of IP-SCC cases, and 35% of SNSCC cases by PCR analysis. Because HPV was not transcriptionally active and had a low viral load in IP, it is still controversial whether HPV DNA is involved in the etiology of IP. On the contrary, HR-HPV infection in IP-SCC and SNSCC accompanied by p16 overexpression was observed in 28.5% and 25% of cases, respectively, suggesting that HR-HPV infection is a crucial factor in sinonasal cancer, consistent with previous studies [14,23,24].

Regarding the impact of HPV type, HPV-16 was the primary type, followed by HPV-33 and HPV-18. The observed HPV types were consistent with the HR-HPV types detected in oropharyngeal carcinoma in our institution [22]. Information on viral load in head and neck cancers is limited. In our previous study of oropharyngeal carcinoma, the viral loads of HPV-16-positive samples ranged from 0.24 to 2.69 × 10^5^ copies/ng DNA (mean 3.49 × 10^4^ and median 3.1 × 10^2^ copies/ng DNA) [25]. In the present study, the viral loads of HPV-16 and -33 in IP-SCC and SNSCC ranged from 0.7 to 1.63 × 10^6^ copies/ng DNA. Although the sample number was limited, the viral loads in IP-SCC and SNSCC were not significantly different from those in oropharyngeal carcinoma. The results for p16 overexpression and HPV viral load suggest a subgroup of HPV-related lesions in IP-SCC and SNSCC.

*EGFR* ex20ins are heterogeneous but comprise mainly in-frame insertions and duplications that cluster between amino acids 762 and 774 [26]. The amino acids changed by ex20ins in the present study were located in the tyrosine kinase domain, which contains the C-helix and the loop following the C-helix. These ex20ins result in the internal rotation of the C-helix, thereby promoting the constitutive activation of EGFR [26]. Qin et al. reported that 2.24% of non-small cell lung cancer cases had unique ex20ins, with A767_V769dup (25.1%) and S768_D770dup (17.6%) as the most prevalent [27]. ex20ins resulting in amino acid changes were observed in up to 50% of IP cases and 57% of IP-SCC cases, and ex20ins were identified only in IP and IP-SCC cases. The most common subtype of ex20ins was S768_D770dup in the IP-SCC group. Although ex20ins are not common in sinonasal cancer (6.15%) [26], IP-SCC has a high incidence of ex20ins (30–90%) compared with other cancers [6,10,28]. In the present study, ex20ins were observed frequently in the IP group, suggesting that they are involved in the etiology of IP.

Y845 is an Src-induced transphosphorylation site on EGFR, which is located in an activation loop that influences kinase activity and is also recognized as a key residue for the oncogenic properties of EGFR [29]. When EGF binds to the extracellular domain of EGFR, the regulatory C-helix rotates from an outward to an inward orientation, leading to the constitutive activation of EGFR [30]. ex20ins has been considered to promote C-helix rotation by pushing the α-C helix, resulting in a conformational change of EGFR in the absence of EGF binding [17,30]. In the present study, ex20ins was located between amino acids 768 and 774 and may induce continuous EGFR activation without ligand binding. The active conformation of the tyrosine kinase domain leads to the autophosphorylation of tyrosine residues (including Y703, Y920, Y992, Y1045, Y1068, Y1086, Y1148, and Y1173) in the intracellular C-terminal tail of EGFR [31]. Autophosphorylation of Y1068 and Y1197 has been reported in ex20ins cases [10,32], which was also observed in our series. However, the most prominent phosphorylation site was Y845 in IP-SCC cases with S768_D770dup. Y845 in EGFR is similarly located in the activation segment of the kinase domain but has distinctive features. Y845 phosphorylation is not mediated by EGFR autophosphorylation but is co-mediated by c-Src [33]. c-Src binds to EGFR and phosphorylates its tyrosine residues to induce cell transformation and carcinogenesis [34]. The conformational change of the kinase domain induced by S768_D770dup allows c-Src access to Y845, which may lead to EGFR phosphorylation (Figure 2 and Figure 3) [35]. The present report is the first to demonstrate Y845 phosphorylation in IP and IP-SCC cases with EGFR ex20ins. In addition, HPV-related tumors, IP-SCC-3, SNSCC-2, and oropharyngeal carcinoma showed Y845 phosphorylation despite the lack of ex20ins. Although the cause of Y845 phosphorylation in HPV-related carcinomas remains unclear, it might be due to carcinogenesis induced by HPV infection.

In the present study, active HR-HPV infection was observed in 28.5% of IP-SCC and 25% of SNSCC but not in IP and sinusitis, while sinusitis samples were different from the nasal mucosa in the healthy subjects. On the contrary, ex20ins was observed in 45% of IP, 28.5% of IP-SCC, and 0% of SNSCC and sinusitis. IP and IP-SCC are diagnosed by histological findings. Thus, these phenomena reflect that IP-SCC might be a multifactorial disease. The active HR-HPV infection and ex20ins might be responsible for the pathogenesis of half of IP-SCC cases from the present results. Although case numbers were limited, patients with active HR-HPV infection did not have ex20ins, except for one IP-SCC case (IP-SCC-1 in Table 3). The finding is in accordance with the previous reports [6,9,17]. Further investigation is needed to understand IP-SCC etiology fully, including the roles of HR-HPV and *EGFR* mutations.

## 5. Conclusions

HPV infection status and *EGFR* ex20ins were investigated in IP, IP-SCC, and SNSCC. HR-HPV infection in IP was observed with a low viral load and was not transcriptionally active. On the contrary, IP-SCC and SNSCC had transcriptionally active infections with high viral load. These results suggest that HPV integration may occur during persistent infection in IP and eventually induce malignant transformation to IP-SCC. *EGFR* ex20ins was frequently detected in IP and IP-SCC but not in SNSCC and chronic sinusitis. Since *EGFR* ex20ins in IP and IP-SCC induced EGFR phosphorylation and activated the PI3K/AKT/mTOR activation, IP and IP-SCC might have a different etiology from SNSCC. The present report is the first to demonstrate EGFR Y845 phosphorylation in IP and IP-SCC cases with EGFR ex20ins. Patients with active HR-HPV infection did not have ex20ins, except for one IP-SCC case. Since IP-SCC is histologically determined, further investigation is needed to fully understand IP-SCC etiology.

## Figures and Tables

**Table 2:** HPV infection status.

Patient	PCR	ISH	HPV Type	Viral Load (Copies/ng DNA)	*E2/E6*	p16 (+)	HPV-Related Tumor/Cancer
IP-1	(+)		16	n.d.	n.d.	Negative	No
IP-5	(+)		16	n.d.	n.d.	Negative	No
IP-11	(+)		33	n.d.	n.d.	Negative	No
IP-12	(+)		11			Negative	No
IP-13	(+)		6			Negative	No
IP-SCC-1	(+)		16	30.5	0.65	Negative	No
IP-SCC-3	(+)		18	2628.0	0.00	Positive	Yes
IP-SCC-4	(+)	(+)	33	n.d.	n.d.	Positive	Yes
IP-SCC-5	(+)		11			Negative	No
SNSCC-2	(+)		16	141.6	0.05	Positive	Yes
SNSCC-3	(+)		33	1,630,004.7	0.83	Positive	Yes
SNSCC-4	(+)		16	3379.2	0.03	Positive	Yes
SNSCC-5	(+)	(+)	n.det.	n.d.	n.d.	Positive	Yes
SNSCC-6	(+)	(+)	n.det	n.d.	n.d.	Positive	Yes
SNSCC-7	(+)		16	1.58	0.09	Negative	No
SNSCC-8	(+)		16	0.7	0.11	Negative	No

HPV, human papillomavirus; ISH, HPV-DNA in situ hybridization; IP, inverted papilloma; IP-SCC, inverted papilloma, and squamous cell carcinoma; n.d., not detected; n.det., could not be determined; p16 (+), p16 overexpression; SNSCC, sinonasal squamous cell carcinoma.

**Table 3:** EGFR mutations and HPV infection in individual patients.

Patient	EGFR Exon 20 Insertion/Duplication	EGFR Exon 20 Substitution	HPV DNA	p16 (+)	Tumor Location	Prognosis
IP-1	**N771_H773dup**	**Q787L**	**P (HPV-16)**	N	M	NR (54 mo.)
IP-2	**D770_** **N771insGF**	**C781R, Y801H**	N	N	M	NR (117 mo.)
IP-3	**H773_V774dup**	**N808D**	N	N	M	NR (24 mo.)
IP-4	**D770_** **N771insGF**	**V819A**	N	N	M	NR (24 mo.)
IP-5	**H773_V774dup**	wt	**P (HPV-16)**	N	M	NR (69 mo.)
IP-6	**S768_D770dup**	wt	N	N	E	R (12 mo.)
IP-7	**N771_H773dup**	wt	N	N	M	NR (9 mo.)
IP-8	**N771_H773dup**	wt	N	N	S	NR (19 mo.)
IP-9	**H773_** **V774insTH**	wt	N	N	M	NR (37 mo.)
IP-10	wt	**Q787X**	N	N	M	NR (9 mo.)
IP-11	wt	wt	**P (HPV-33)**	N	M	NR (31 mo.)
IP-12	wt	wt	**P (HPV-11)**	N	E	NR (55 mo.)
IP-13	wt	wt	**P (HPV-6)**	N	E	NR (13 mo.)
IP-14	wt	wt	N	N	M	NR (34 mo.)
IP-15	wt	wt	N	N	M	NR (69 mo.)
IP-16	wt	wt	N	N	E	NR (24 mo.)
IP-17	wt	wt	N	N	M	NR (64 mo.)
IP-18	wt	wt	N	N	E	NR (4 mo.)
IP-19	wt	wt	N	N	M	NR (9 mo.)
IP-20	wt	wt	N	N	M	NR (12 mo.)
IP-SCC-1	**S768_D770dup**	**D800G**	**P (HPV-16)**	N	M	NR (36 mo.)
IP-SCC-2	**S768_D770dup**	**L782F**	N	N	E	DOD (7 mo.)
IP-SCC-3	wt	**S811P**	**P (HPV-18)**	**P**	E	NR (54 mo.)
IP-SCC-4	wt	**T790A**	**P** **(type N/A)**	**P**	S	DOD (6 mo.)
IP-SCC-5	wt	wt	**P (HPV-11)**	N	M	NR (72 mo.)
IP-SCC-6	wt	wt	N	N	M	NR (116 mo.)
IP-SCC-7	wt	wt	N	N	M	AWD (14 mo.)
SNSCC-1	wt	**M793V**	N	N	M	DOD (21 mo.)
SNSCC-2	wt	wt	**P (HPV-16)**	**P**	NC	NR (10 mo.)
SNSCC-3	wt	wt	**P (HPV-33)**	**P**	NC	NR (54 mo.)
SNSCC-4	wt	wt	**P (HPV-16)**	**P**	M	NR (69 mo.)
SNSCC-5	wt	wt	**P** **(type N/A)**	**P**	M	NR (24 mo.)
SNSCC-6	wt	wt	**P** **(type N/A)**	**P**	M	NR (95 mo.)
SNSCC-7	wt	wt	**P (HPV-16)**	N	M	NR (66 mo.)
SNSCC-8	wt	wt	**P (HPV-16)**	N	E	NR (71 mo.)
SNSCC-9	wt	wt	N	N	M	IDD (4 mo.)
SNSCC-10	wt	wt	N	N	M	NR (62 mo.)
SNSCC-11	wt	wt	N	N	M	NR (68 mo.)
SNSCC-12	wt	wt	N	N	M	DOD (21 mo.)
SNSCC-13	wt	wt	N	N	M	DOD (9 mo.)
SNSCC-14	wt	wt	N	N	M	NR (40 mo.)
SNSCC-15	wt	wt	N	N	M	NR (65 mo.)
SNSCC-16	wt	wt	N	N	M	DOD (7 mo.)
SNSCC-17	wt	wt	N	N	M	NR (65 mo.)
SNSCC-18	wt	wt	N	N	M	NR (73 mo.)
SNSCC-19	wt	wt	N	N	M	NR (70 mo.)
SNSCC-20	wt	wt	N	N	M	DOD (28 mo.)
CRS-1	wt	wt	N	N		
CRS-2	wt	wt	N	N		
CRS-3	wt	wt	N	N		
CRS-4	wt	wt	N	N		
CRS-5	wt	wt	N	N		
CRS-6	wt	wt	N	N		

AWD, alive with disease; CRS, chronic rhinosinusitis; DOD, dead of disease; E, ethmoid sinus; HPV, human papillomavirus; IDD, intercurrent disease death; IP, inverted papilloma; IP-SCC, inverted papilloma, and squamous cell carcinoma; M, maxillary sinus; mo., months; mo., follow-up months; N, negative; NC, nasal cavity; NR, no recurrence/alive without disease; P, positive; p16 (+), p16 overexpression; S, sphenoid sinus; SNSCC, sinonasal squamous cell carcinoma; wt, wild type.

## Data Availability

The datasets generated and/or analyzed during the present study have not been made publicly available. However, data can be made available from the corresponding author upon reasonable request.

## Supplementary Materials

The following supporting information can be downloaded at https://www.mdpi.com/article/10.3390/jpm13040657/s1: Figure S1. Localization of *EGFR* ex20ins between amino acids 768 and 774 in IP and IP-SCC; Table S1. Primers used for HPV infection analysis and Sanger sequencing of *EGFR* exon 20; Table S2. Relationship between p16 immunoreactivity and HR-HPV in IP; and Table S3. EGFR amino acid insertions/duplications and substitutions.
