# Supplementary material for: Human Papillomavirus Infection and EGFR Exon 20 Insertions in Sinonasal Inverted Papilloma and Squamous Cell Carcinoma
Source: J Pers Med. 2023 Apr 11;13(4):657. doi: 10.3390/jpm13040657 (PMC10143312; doi:10.3390/jpm13040657)
Supplement: Supplementary file 1 [file jpm-13-00657-s001.zip › jpm-2339772-supplementary.docx]

Supplementary Materials for

Human papillomavirus infection and *EGFR* exon 20 insertions in sinonasal inverted papilloma and squamous cell carcinoma

Hitoshi Hirakawa ^1**^, Taro Ikegami ^1**^, Norimoto Kise ^1^, Hidetoshi Kinjyo ^1^, Shunsuke Kondo ^1^, Shinya Agena ^1^, Narumi Hasegawa ^1^, Junko Kawakami ^1^, Hiroyuki Maeda ^1^ and Mikio Suzuki ^1,^*

**Contents**

**Supplementary Methods**

**Figure S1.** Localization of *EGFR* ex20ins between amino acids 768 and 774 in IP and IP-SCC.

**Table S1.** Primers used for HPV infection analysis and Sanger sequencing of *EGFR* exon 20.

**Table S2.** Relationship between p16 immunoreactivity and HR-HPV in IP.

**Table S3.** EGFR amino acid insertions/duplications and substitutions.

**Supplementary Methods**

**1. HPV infection analysis**

HPV infection was analyzed by PCR using fresh frozen samples, following p16 overexpression analysis with immunohistochemistry using FFPE samples. DNA was extracted from the fresh frozen samples using a Gentra Puregene Tissue Kit (Qiagen, Germantown, MD), and the presence and integrity of DNA were confirmed in all samples. If there were insufficient fresh frozen samples for PCR, HPV-DNA ISH and PCR were carried out using FFPE sections and DNA extracted from FFPE samples, respectively. The DNA extraction areas were required to contain at least 30% tumor nuclei of total cell nuclei for IP, IP-SCC, and SNSCC cases.

FFPE samples were used in histologic studies for hematoxylin and eosin staining, p16 immunohistochemistry, and HPV-DNA ISH. For HPV infection analysis, DNA from FFPE samples, macrodissected from 10-µm sections, was extracted using a GeneRead DNA FFPE Kit (Qiagen, Gaithersburg, MD). Viral load was measured, and the physical status of HPV-16-infected samples was clarified as described previously.^1^ Primers for PCR assays in the present study are listed in Table S1.

Furthermore, new quantitative real-time PCR assays for the *E6* and *E2* genes of HPV-18, -33, and -52 were established. To make standard DNA for each HPV subtype, the entire length of *E6* and partial length of *E2* were amplified by PCR (primers are listed in Table S1) using HPV-18, -33, or -52-infected DNA samples as templates. PCR was performed with F and R primers (0.24 μM each, Table S1) in a volume of 12.5 μL containing 6.3 μL GoTaq® Green Master Mix (Promega, Madison, WI) and 10 ng genomic DNA. The PCR profile was as follows: denaturation at 95°C for 3 min, followed by 35 cycles at 95°C for 30 s, 60°C for 30 s, and 72°C for 30 min, and a final extension at 72°C for 1 min. PCR fragments of the expected size were purified by the Wizard SV Gel and PCR Clean-Up System (Promega). The PCR fragments of *E6* of HPV-18 and -33 were digested with *Eco*RI and *Hpa*I and then subcloned into the *Eco*RI and *Eco*RV restriction sites of pcDNA3.1 (+) (Invitrogen, Carlsbad, CA). The entire length of the HPV-52 *E6* gene was synthesized by Eurofins Genomics Co. (Tokyo, Japan) according to the DNA sequence reported in the PapillomaVirus Episteme database (https://pave.niaid.nih.gov/locus_viewer?seq_id=HPV52REF) and then subcloned into pcDNA3.1 (+) as described above. The PCR products of *E2* of HPV-33 and -52 were subcloned into pGEM-T Easy (Promega, Madison, WI). The plasmids were sequenced with an ABI PRISM 3130xl Genetic Analyzer (Applied Biosystems, Foster City, CA). Then, the plasmids were serially diluted as mentioned earlier and the HPV-18 *E2* plasmid (p2092), which was a gift from Peter Howley (Addgene plasmid #10876; https://www.addgene.org/10876/; accessed on June 9, 2022), was used to establish standard curves (1.0 × 10^3^–1.0 × 10^7^ copies/2 μL). Quantitative real-time PCR was performed with the CFX96 Touch™ Real-Time PCR Detection System (Bio-Rad, Hercules, CA). The PCR reaction mixture (10 μL) contained 0.2 μM primers, 5.0 μL SYBR Premix Ex Taq^TM^ II (Tli RNaseH plus; Takara, Otsu, Japan), and 2.0 μL standard plasmid DNA or genomic DNA (30 ng). The PCR profile was as follows: 95°C for 30 s followed by 40 cycles at 95°C for 5 s and 60°C for 30 s. Specific amplification of each HPV DNA was verified by melting curve analysis and gel electrophoresis of the products.

An external standard curve was created using serial dilutions (0.3, 3, 30, and 300 ng) of human genomic placental DNA (Sigma-Aldrich, St. Louis, MO; Merck KGaA, Darmstadt, Germany) for cellular DNA quantification, and β-globin was amplified as an internal control. Viral DNA load was assessed by calculating *E6* copy number.^1^ The total *E6* copy number in 1 ng cellular DNA was determined. Then, the ratio of the *E2* copy number/total *E6* copy number was calculated. An *E2/E6* ratio ≥ 1 denotes the predominance of the episomal form, whereas 0 < *E2/E6* ratio < 1 demonstrates a mix of both integrated and episomal forms. A ratio of 0 indicates the presence of the integrated form only.

**2. Supplementary Methods for *EGFR* mutation analysis**

EGFR exon 20 mutation analysis was performed using Sanger sequencing (primers in Table S1). Western blotting and immunohistochemistry were used to analyze the effects of the detected EGFR mutations on downstream proteins.

***2.1. Sanger sequencing of EGFR exon 20***

The PCR profile was as follows: denaturation at 95°C for 3 min, followed by 35 cycles at 95°C for 30 s, 60°C for 30 s, and 72°C for 30 min, and a final extension at 72°C for 1 min. PCR fragments of the expected size were purified as described above. The purified PCR products were cloned into pGEM-T Easy (Promega) and sequenced as described above

***2.2. Screening of EGFR phosphorylation sites***

Briefly, IP-SCC tissues were homogenized and sonicated in ice-cold 1× Cell Lysis Buffer, including 1× Proteinase Inhibitor Cocktail, 1× Phosphatase Inhibitor Cocktail Set I, and 1× Phosphatase Inhibitor Cocktail Set II (RayBiotech, Inc., Peachtree Corners, GA). Lysate protein concentration was measured using a DC Protein Assay Kit (Bio-Rad). Lysate protein (1.0 mL of 200 µg/mL) was input into the Antibody Array. The images were scanned using the ChemiDoc XRS+ System (Bio-Rad).

***2.3. Western blot analysis***

Each protein sample for western blotting was prepared at a concentration of 12 µg/21 µL in 62.5 mM Tris-HCl (pH 6.8), 2% SDS, 10% glycerol, 6% mercaptoethanol, and 0.01% bromophenol blue, and boiled for 5 min. The samples were electrophoresed in 7.5% or 12.5% polyacrylamide-SDS gels and transferred to polyvinylidene difluoride (PVDF) membranes. The membranes were blocked in PVDF Blocking Reagent for Can Get Signal^®^ (Toyobo Co., Ltd., Osaka, Japan) for 30 min and incubated with diluted primary antibodies. The membranes were appropriately incubated with horseradish peroxidase-conjugated secondary antibodies against rabbit IgG (1:2,000 dilution, #7077; Cell Signaling Technology [CST], Danvers, MA), rat IgG (1:5,000 dilution, A9037; Sigma-Aldrich), or mouse IgG (1:2,000 dilution, #1706516; Bio-Rad) for 1 h at room temperature. Bands were visualized using Clarity Western ECL Substrate (Bio-Rad) and photographed with the ChemiDoc XRS+ System.

The antibodies and their dilutions used in western blotting were as follows: EGFR rabbit monoclonal (1:1,000 dilution, D38B1; CST); p-EGFR (Y845) rabbit polyclonal (1:1,000 dilution, 44-784G; Invitrogen); p-EGFR (Y1068) rabbit monoclonal (1:1,000 dilution, D7A5; CST); p-EGFR (Y1086) rabbit polyclonal (1:1,000 dilution, 44-790G; Invitrogen); p-EGFR (Y1197) rat monoclonal (1:5,000 dilution, clone #869286; Novus Biologicals, Littleton, CO); Akt rabbit monoclonal (1:1,000 dilution, C67E7; CST); p-AkT (S473) rabbit monoclonal (1:1,000 dilution, D9E; CST); 4E-BP1 rabbit monoclonal (1:1,000 dilution, 53H11; CST); p-4E-BP1 (T37/46) rabbit monoclonal (1:1,000 dilution; CST); STAT3 mouse monoclonal (1:1,000 dilution, 124H6; CST); p-STAT3 (Y705) rabbit monoclonal (1:1,000 dilution, D3A7; CST); p16INK4a mouse monoclonal (1:2,000 dilution, JC8; Santa Cruz Biotechnology, Dallas, TX); and pan-actin rabbit polyclonal (1:1,000 dilution, 4968; CST).

***2.4. Immunohistochemistry for EGFR and p-EGFR***

Epitope retrieval was achieved by heating the sections at 100°C for 10 min in 1 mM EDTA buffer (pH 8.0). Endogenous peroxidase activity was quenched by incubating the sections in 0.3% H_2_O_2_ in methanol for 20 min at room temperature. Immunolabeling was visualized by incubation in 3-3′-diaminobenzidine, and stained slides were counterstained with hematoxylin.

**Supplementary References**

1. Ikegami T, Uehara T, Deng Z, et al. Detection of human papillomavirus in branchial cleft cysts. *Oncol. Lett.* 2018, *16*, 1571-1578.


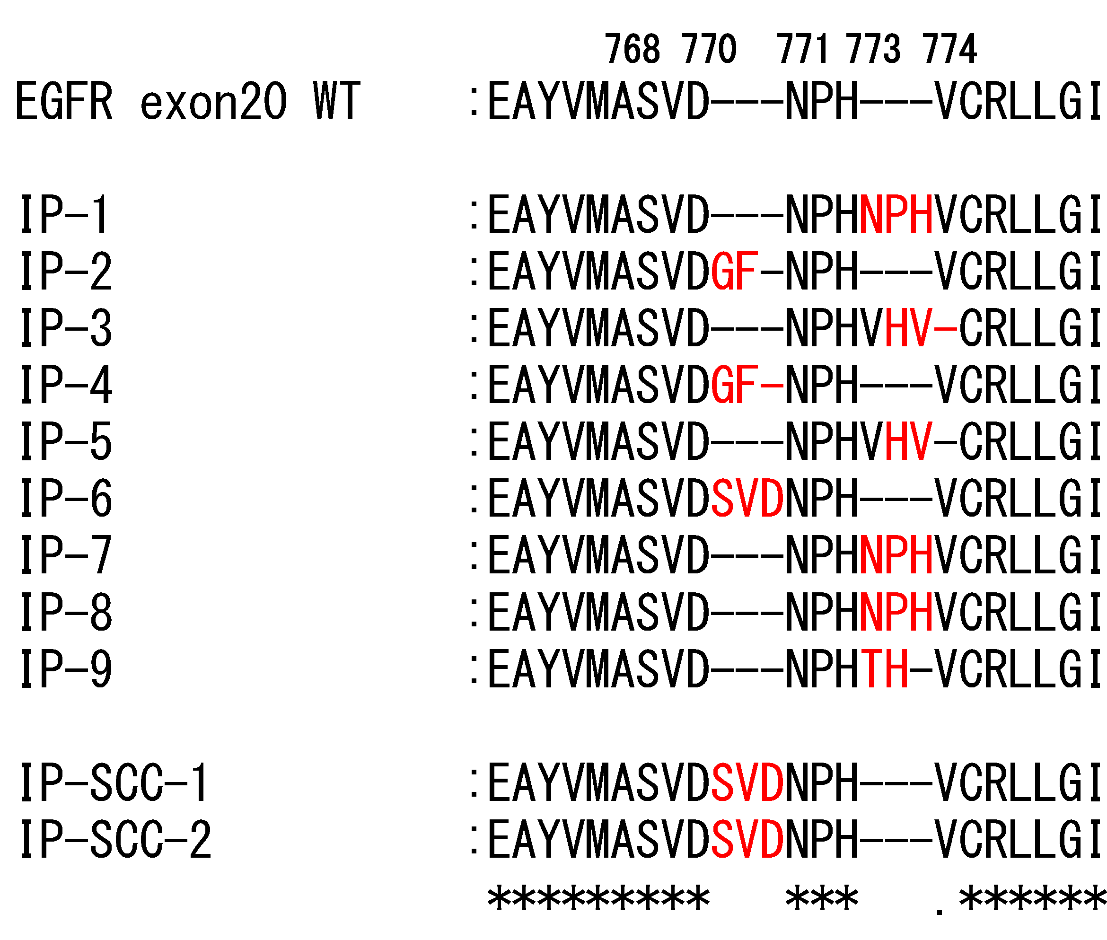


**Figure S1. Localization of *EGFR* ex20ins between amino acids 768 and 774 in IP and IP-SCC.**

Red letters indicate amino acid insertions or duplications. Asterisk (*) and dot (.) indicate completely and almost identical residues, respectively.

**Table S1. Primers used for HPV infection analysis and Sanger sequencing of *EGFR* exon 20.**

| **Cloning primers for standard DNA** | | | **Sequence (5′–3′)** | | | |
| --- | --- | --- | --- | --- | --- | --- |
| HPV-18 *E6*-F | | | AAAGAATTCACCACCATGGCGCGCTTTGAGGAT | | | |
| HPV-18 *E6*-R | | | AAAGTTAACTACTTGTGTTTCTCTGCGTCGTT | | | |
| HPV-33 *E6*-F | | | AAAGAATTCACCACCATGTTTCAAGACACTGAGGAAAAACC | | | |
| HPV-33 *E6*-R | | | AAAGTTAACCAGTGCAGTTTCTCTACGTCGG | | | |
| HPV-33 *E2*-F | | | TGGAAACTGATACGCATGGA | | | |
| HPV-33 *E2*-R | | | GCAATGTCCATTGGCTTGTA | | | |
| HPV-52 *E2*-F | | | AGAAACGACGACGACCAGAC | | | |
| HPV-52 *E2*-R | | | TCAGTTGCAGTGACGAGTCC | | | |
|  |  |  |  |  |  |  |
| **Real-time PCR primers** | | | **Sequence (5′–3′)** | | | |
| HPV-18 *E6*-qPCR-F | | | TGAAAAACGACGATTTCACAAC | | | |
| HPV-18 *E6*-qPCR-R | | | TACTTGTGTTTCTCTGCGTCGT | | | |
| HPV-18 *E2*-qPCR-F | | | GCAGCTACACCTACAGGCAAC | | | |
| HPV-18 *E2*-qPCR-R | | | CGCTATGTTTTCGCAATCTGT | | | |
| HPV-33 *E6*-qPCR-F | | | AAACCACGAACATTGCATGA | | | |
| HPV-33 *E6*-qPCR-R | | | TCCAAATGGATTTCCCTCTCT | | | |
| HPV-33 *E2*-qPCR-F | | | TGGAAACTGATACGCATGGA | | | |
| HPV-33 *E2*-qPCR-R | | | GCAATGTCCATTGGCTTGTA | | | |
| HPV-52 *E6*-qPCR-F | | | TCAAACGCCATTATGTCCTG | | | |
| HPV-52 *E6*-qPCR-F | | | GGGGTCTCCAACACTCTGAA | | | |
| HPV-52 *E2*-qPCR-F | | | AGAAACGACGACGACCAGAC | | | |
| HPV-52 *E2*-qPCR-R | | | TCAGTTGCAGTGACGAGTCC | | | |
|  |  |  |  |  |  |  |
| **Cloning primers for *EGFR* exon 20** | | | **Sequence (5′–3′)** | | | |
| *EGFR* exon 20-F | | | CCACCATGCGAAGCCACACTGA | | | |
| *EGFR* exon 20-R | | | TCCTTATCTCCCCTCCCCGTATCTC | | | |

Cloning primers used for standard DNA of HPV-33 *E2* and HPV-52 *E2* are the same as the real-time PCR primers for HPV-33 *E2* and HPV-*52* *E2*, respectively.

**Table S2. Relationship between p16 immunoreactivity and HR-HPV in IP.**

|  | <40% | 40%–75% | ≥75% |
| --- | --- | --- | --- |
| Positive cell counts in p16 IHC | 8 (40%) | 7 (35%) | 5 (25%) |
| Positive HR-HPV DNA by PCR | 1 | 1 | 1 |

HR-HPV, high-risk human papillomavirus; IHC, immunohistochemistry; IP, inverted papilloma.

**Table S3. EGFR amino acid insertions/duplications and substitutions.**

| Group | Insertion/duplication | Substitution | Number of cases  (%) | |
| --- | --- | --- | --- | --- |
| IP | positive | negative | 5 | 25 |
| *n* = 20 |  | positive | 4 | 20 |
|  | negative | negative | 10 | 50 |
|  |  | positive | 1 | 5 |
| IP-SCC | positive | negative | 0 | 0 |
| *n* = 7 |  | positive | 2 | 29 |
|  | negative | negative | 3 | 42 |
|  |  | positive | 2 | 29 |
| SNSCC | positive | negative | 0 | 0 |
| *n* = 20 |  | positive | 0 | 0 |
|  | negative | negative | 19 | 95 |
|  |  | positive | 1 | 5 |
| CRS | positive | negative | 0 | 0 |
| *n* = 6 |  | positive | 0 | 0 |
|  | negative | negative | 6 | 100 |
|  |  | positive | 0 | 0 |

CRS, chronic rhinosinusitis; IP, inverted papilloma; IP-SCC, inverted papilloma and squamous cell carcinoma; SNSCC, sinonasal squamous cell carcinoma.
